# Oral Contraceptive Knowledge Among Adolescents and Young Women

**DOI:** 10.3390/pharmacy14010030

**Published:** 2026-02-05

**Authors:** Nga-Weng (Ivy) Leong, Marie Barnard, Meagen Rosenthal, Erin Holmes

**Affiliations:** Department of Pharmacy Administration, School of Pharmacy, University of Mississippi, University, MS 38677, USA; nleong@go.olemiss.edu (N.-W.L.); mbarnard@olemiss.edu (M.B.); mmrosent@olemiss.edu (M.R.)

**Keywords:** oral contraceptives, adolescents and young adults, contraceptive knowledge

## Abstract

This study aims to describe oral contraceptive knowledge among adolescents and young women, and to examine individuals’ characteristics associated with oral contraceptive knowledge. A cross-sectional survey was administered using an online panel. Females aged 16 to 24 were recruited. Oral contraceptive knowledge was measured using nine items with six domains, including oral contraceptive use, efficacy, indication, mechanism of action, risks, and side effects. A summated score was created, with a score of 9 indicating highest level of knowledge. Multivariable regression was used to examine significant socio-demographics and clinical characteristics. Among the 700 included responses, largest proportion of respondents were White (45.43%) and were covered by public insurance (43.14%). A total of 446 (63.71%) respondents expressed at least slight interest in using over-the-counter oral contraceptives. Overall, the mean score of knowledge was 4.08 out of 9. Most did not correctly answer questions about side effects, the mechanism of action and appropriate use. Similar patterns were observed among those who were interested in over-the-counter oral contraceptives (mean = 4.11). Adolescents and young women had a low level of oral contraceptive knowledge. With a high proportion of individuals interested in over-the-counter oral contraceptives, additional information support is needed to support informed contraception choice and use.

## 1. Introduction

Oral contraceptive pills (OCPs) are the most commonly used method of contraception among young women aged 15 to 19 in the United States, with 14.2% of them using OCPs [1]. In 2023, the first over-the-counter (OTC) OCPs (active ingredient: 0.075 mg norgestrel, brand name: Opill^®^; Perrigo, Allegan, MI, USA) were approved in the US [2]. This OTC OCP is proven to be safe and effective in preventing unintended pregnancy when used as directed [3,4]. Over-the-counter OCPs are expected to improve contraception access, especially for individuals without insurance, those of a younger age, and those living in rural areas [2,5]. OCPs are safe and effective among adolescents, while contraindications are rare among adolescents [5]. Since OCPs became available OTC in stores in April 2024, there has been increased contraceptive uptake among young women [2]. With the anticipation of a higher uptake and documented interest among young populations, knowledge about OCPs becomes particularly important to facilitate appropriate use and support contraception autonomy.

Adolescents and young adults choosing a suitable contraceptive method should consider different aspects of contraceptives, including the benefits and side effects, comparative effectiveness among potential options, and awareness of the importance of protection against sexually transmitted diseases [6]. Such contraceptive knowledge may influence contraceptive behavior, such as willingness to use [7] and adherence [8], thus enhancing the effectiveness of contraceptives in preventing unintended pregnancies among young populations. However, it has been shown that overall contraceptive knowledge is consistently low among adolescents and young adults [9,10,11,12,13]. These studies were able to capture knowledge and attitudes across a variety of contraceptives. As such, there remains a gap in understanding and assessing the level of OCP knowledge among young populations.

Given the various contraceptive choices available, adolescents and young adults may find it overwhelming to find the best method [6,14]. During their decision-making process, information about birth control options is one of the critical components [15]. In addition, young women also tend to make contraceptive decisions depending on their contraceptive values and preferences, which are mostly based on their contraceptive knowledge [16]. Therefore, given the continuous improvement in OCP accessibility [17] and a consistently low level of overall contraceptive knowledge among young populations, this study aims to describe the level of OCP knowledge among adolescents and young women, and to explore the characteristics associated with OCP knowledge.

## 2. Materials and Methods

A cross-sectional online survey was administered in April 2025 using a Qualtrics panel. Data included in this study are a part of a larger study that examined the willingness to approach pharmacists for OTC OCP information. Females aged 16 to 24 in the US were recruited using an online panel from Qualtrics, with a balance of adolescents (16–17) and young adults (18–24). The anonymous online-only survey was distributed by Qualtrics to potential participants of their online panel. Qualtrics used nonprobability convenience sampling to recruit their survey panel members who meet the inclusion criteria. Data were collected during April 2025. Approval from the Institutional Review Board of the investigators’ university was obtained before data collection. This study was conducted in accordance with the Strengthening the Reporting of Observational Studies in Epidemiology (STROBE) reporting guidelines [18].

Socio-demographic characteristics were collected, including age, race, residential state, rurality, insurance status, highest education attainment, socioeconomic status [19], health literacy [20], and digital health literacy [21]. We also collected several reproductive-related variables, including previous use of a contraception method, whether the participants were currently sexually active, and contraception concern [22]. Interest in using OTC OCPs was collected using a 5-point scale from “*not interested at all*” to “*extremely interested*”. Individuals were identified as being interested in using OTC OCPs if they responded “*slightly interested”, “moderately interested”, “very interested”,* or *“extremely interested*”. Characteristics were described for the overall sample, and stratified by their OTC OCP interest.

Oral contraceptive knowledge was measured with nine items across six domains. Given the lack of a validated standardized tool for oral contraceptive knowledge, the items were developed and guided by the previous literature [13]. According to Hall, previous questionnaires had frequently evaluated contraceptive knowledge on domains including how to use OCPs, efficacy, indication, risk, mechanism of action, and how to manage side effects [13]. The items were developed and reviewed by authors through a literature review on these knowledge domains and previous questionnaires to ensure the face validity. The knowledge items can be found in Table A1. A summated score was calculated, ranging from 0 to 9. A higher score indicates a higher level of OCP knowledge. We reported item score, total score and proportion of getting all correct answers in each domain for the overall sample and stratified by their OTC OCP interest. We did not provide the unadjusted significance for each item or domain between the two groups as such comparisons would be underpowered and confounded by individual characteristics.

Careless responses were identified by straight-lining among main constructs used in the larger study and were excluded from this analysis. The survey was distributed to 1076 individuals, and the response rate was 70.35% (*n* = 757). Out of 757 responses, 57 responses were excluded due to straight-lining. Exploratory multivariable regression with listwise deletion was used to examine any significant associations between individual characteristics and total knowledge score. Data management and analysis were conducted in SAS version 9.4 (SAS Institute, Cary, NC, USA).

## 3. Results

Out of the 700 responses included, largest proportion of respondents were White (45.43%) and were living in the southern states (44.71%). Around 40.14% were under their parent’s/guardian’s health plan or had private insurance coverage, and 43.14% were covered by public insurance. Around 33.14% of respondents had used some type of contraceptive methods. Around 23% of respondents did not have any previous interactions with pharmacists or pharmacy technicians. Overall, they reported a moderate level of interest in using OTC OCPs, with a mean score of 2.28 out of 5. A total of 446 respondents expressed at least slight interest in using OTC OCPs. A higher proportion of individuals who were interested in OTC OCP had some college or bachelor’s degree than that among those who were not interested (24.22% vs. 19.69%, *p* = 0.021). There was also a higher proportion of individuals who were interested in OTC OCP use that had previous contraception use. The detailed characteristics are presented in Table 1.

Overall, the mean score of OCP knowledge (range: 0–9) was 4.08, with a standard deviation of 1.89. Among all respondents, only 65 (9.29%) respondents answered the questions about side effects correctly. Less than half of respondents were able to correctly answer questions about the mechanism of action (14.29%), appropriate use (38%), the benefits of using oral contraceptives (46%), and the efficacy of these medications (46.86%), as described in Table 2 and Table 3. The overall knowledge level (mean = 4.11) and levels at each domain among those who reported interest in using OTC OCPs were similar to the overall sample.

Exploratory multivariable regression found that interest in OTC OCPs was not significantly associated with OCP knowledge (0.014, *p* = 0.916). African Americans (−0.591, *p* < 0.001) and Hispanic individuals (−0.619, *p* = 0.002) had significantly lower OCP knowledge, compared to White individuals (Table 4). Individuals covered by public insurance (−0.350, *p* = 0.019) or who were uninsured (−0.594, *p* = 0.002) also had significantly lower OCP knowledge, compared to those with private insurance. The frequency of interaction with pharmacists was associated with a decrease in OCP knowledge (−0.211, *p* = 0.001). Lastly, higher health literacy (0.125, *p* < 0.001) and higher contraception concern (0.293, *p* = 0.002) were also associated with higher OCP knowledge.

## 4. Discussion

This study found that adolescents and young women overall had a low level of OCP knowledge. Individuals without previous contraceptive use had a lower level of knowledge. However, interest in OTC OCPs was not associated with OCP knowledge. A large proportion of adolescents and young women were not able to provide correct answers about identifying and managing side effects, how the medication works in the body, and what to do after missing doses. Our findings align with most studies investigating contraceptive knowledge, collectively finding a low level of contraceptive knowledge [7,9,10,11,12,23,24]. In particular, most respondents were not fully aware of what the side effects of OCPs are and how to manage them. These findings supplement those from existing qualitative studies that a lack of knowledge regarding side effects was common among young populations [11,12]. This gap in knowledge is particularly concerning because previous research has found that young females were reluctant to use OTC OCPs based on these misconceptions (e.g., OTC OCPs were less effective) or misunderstanding side effects, such as believing that OCPs can cause cancer or long-term infertility [11]. While evidence from a label comprehension study has shown the ability to appropriately use OTC OCPs after reading the medication label [4], hesitancy to use OTC OCPs may have occurred due to misconceptions about long-term risks among adolescents [11]. Moreover, a study that conducted qualitative interviews revealed the participants had fear and anxiety, as well as a lack of understanding how contraceptives work in their bodies [12]. Likewise, our study found that many young adults lack an understanding of the mechanism of action of OCPs. Nonetheless, young populations were generally open to additional information and would appreciate more clear and concise information about effectiveness and side effects [11,12,25]. Although the contraindications among adolescents are rare and OCPs are seen to be safe among younger individuals when used as directed [5], our findings serve as another reminder of the additional informational support needed by younger individuals to ensure appropriate use [26] and support their contraceptive autonomy [27].

Various forms of informational intervention for young adults can be considered. First, pharmacists are easily accessible and are in an ideal position to serve as a OCP information resource, given that OCPs are medications regardless of their OTC status [28]. Interestingly, more interaction with pharmacists was associated with lower OCP knowledge in our study. However, the interaction may not translate to communication regarding contraceptives. Future qualitative research can examine the information exchange and prevalence of medication discussed during the interaction with pharmacists. Nonetheless, pharmacists can provide easily accessible OCP information (e.g., leaflets or infographics) directly to young individuals who are interested in using OTC OCPs. Given that 23% of respondents in our study had never had an interaction with a pharmacist, this approach may not be optimal [5]. For OTC OCPs, pharmacists need to make individuals aware they can approach the pharmacist for information. This may be done via signage or other advertising materials. However, under the OTC self-selection process, this may require additional effort from pharmacists or active help-seeking behavior by younger individuals. Considering this shortcoming, educational interventions that do not require the presence of any healthcare provider should be prioritized, such as content on digital platforms. Most intervention studies that involved the provision of information have found significant improvements in contraceptive knowledge [29,30]. Younger populations may not prefer in-person interaction, with the additional concern of privacy and contraception being a sensitive topic [16]. Future research should identify an optimal form (e.g., written, video) and channel of dissemination (e.g., via app, online website) that can simultaneously allow interactive learning, as well as being acceptable to adolescents and young adults.

With the introduction of OTC OCPs, young populations may be increasingly exposed to these OCPs amongst the variety of contraceptive options. This study serves as an important contribution to understanding areas that can be enhanced for young populations to execute person-centered contraception autonomy [27]. To support adolescents’ contraception autonomy and decision-making, it would be unrealistic to only mention OCPs amongst the variety of contraceptive options. Decision aids are tools that can inform individuals about their options and facilitate shared decision-making between provider and individuals [31]. Several intervention studies have found improved overall contraceptive knowledge after intervention and thus increased contraceptive preference for long-acting reversible contraceptives [32,33], which are the first-line recommendation among eligible adolescents and young women. These studies were mostly conducted in a clinical setting, which may not fully capture the picture behind those who were not able to access a provider or faced other barriers to access. Nonetheless, our findings can hopefully shed light on the types of information that should be emphasized in contraceptive decision aids to facilitate better understanding of contraception options, especially when facing geographic, financial and insurance barriers [34,35,36].

Most respondents were covered by Medicaid and were living in suburban and rural areas. These individuals may have a higher likelihood of considering and using OTC OCPs, provided that suburban and rural areas are more likely to be contraceptive deserts and Medicaid acceptances varied across these areas [37]. Our findings appear to be an emphasis on information support among individuals that shared similar characteristics, with significantly lower OCP knowledge among individuals covered by public insurance. Pharmacists, especially those who practice in such environments, should seek continuing education on oral contraceptives and other methods to confidently provide education to individuals who are interested in OTC OCPs.

The results reported may not be generalized to national representatives of young populations. However, the findings remain meaningful in that the sample contains a high proportion of individuals covered under Medicaid who are more likely to face barriers to accessing contraception care and utilizing OTC OCPs. As the sample in this study was recruited from an online panel, the finding may not be fully reproducible, and future research should attempt to solidify this finding by using a nationally representative sample. Second, the study could not establish any meaningful relationship with reproductive behavior, such as contraception preference. Future research should examine how the level of OCP knowledge can influence reproductive autonomy among young populations using longitudinal data. Third, readers should be cautioned that knowledge was assessed using a newly created, unvalidated scale. Three questions (questions 3, 5, and 8 in Appendix A) were constructed as negative questions which may have influenced item difficulty. We conducted an item analysis and found that item discrimination coefficients (point biserial correlations) ranged from 0.24 to 0.56, and item difficulty (proportion of respondents who answered each of the items correctly) ranged from 0.31 to 0.62, indicating an overall acceptable item discrimination and item difficulty. Questions may be exhibiting varying levels of difficulty, making it difficult to compare performance among domains of knowledge. Lastly, this study focused on the knowledge of solely oral contraceptives among adolescents and young women. Future research can explore the comparison of their knowledge among contraceptives and other OTC medications.

Despite these limitations, our findings underpin the gap in OCP knowledge among adolescents and young women. Our findings may reflect knowledge among different groups of young population, as a result of the data collection process. Most previous studies recruited participants from clinics and hospitals, which may influence their health literacy due to their increased interaction with providers. Also, our sample included individuals aged 16 to 24, which adds value to the understanding of knowledge among younger individuals.

## 5. Conclusions

Our study found that adolescents and young women had a low level of OCP knowledge. The findings shed light on aspects of OCPs that can be emphasized in contraceptive education, particularly regarding managing side effects, the mechanism of action and appropriate use. Especially under the OTC availability and current dynamic in contraception access, the findings serve as a reminder of the need for additional information support among young populations to support their contraception autonomy.

## Figures and Tables

**Table 1:** Characteristics of included individuals.

Characteristics	All Included Individuals (*N* = 700)	Individuals Who Were Interested in Using Over-The-Counter Oral Contraceptives (*n* = 446) ^a^	Individuals Who Were Not Interested in Using Over-The-Counter Oral Contraceptives (*n* = 254)	*p*-Value ^b^
Age (mean [standard deviation])	18.82 (2.61)	18.90 (2.68)	18.69 (2.49)	0.315
Race				0.524
White	318 (45.43%)	211 (47.31%)	107 (42.13%)	
African American	189 (27%)	113 (25.34%)	76 (29.92%)	
Hispanic	100 (14.29%)	63 (14.13%)	37 (14.57%)	
Other (American Indian or Alaska, Asian, Native Hawaiian or other)	93 (13.29%)	59 (13.23%)	34 (13.39%)	
Region ^c^				0.713
Northeast	103 (14.71%)	62 (13.9%)	41 (16.14%)	
Midwest	144 (20.57%)	97 (21.75%)	47 (18.5%)	
South	313 (44.71%)	200 (44.84%)	113 (44.49%)	
West	137 (19.57%)	87 (19.51%)	50 (19.69%)	
Rurality ^d^				0.382
Urban	157 (22.43%)	102 (22.87%)	55 (21.65%)	
Suburban	344 (49.14%)	226 (50.67%)	118 (46.46%)	
Rural	197 (28.14%)	118 (26.46%)	79 (31.1%)	
Insurance status				0.279
Covered under parent/guardian’s plan or private insurance	281 (40.14%)	189 (42.38%)	92 (36.22%)	
Public insurance	302 (43.14%)	185 (41.48%)	117 (46.06%)	
Uninsured	117 (16.71%)	72 (16.14%)	45 (17.72%)	
Marital status				0.866
Never married	520 (74.29%)	330 (73.99%)	190 (74.8%)	
Married	46 (6.57%)	31 (6.95%)	15 (5.91%)	
Other (divorced, separated, widowed, prefer not to say)	134 (19.14%)	85 (19.06%)	49 (19.29%)	
Highest education attained				0.021
High school or less	479 (68.43%)	303 (67.94%)	176 (69.29%)	
Some college or Bachelor degree	158 (22.57%)	108 (24.22%)	50 (19.69%)	
Advanced degree	19 (2.71%)	15 (3.36%)	4 (1.57%)	
Prefer not to say	44 (6.29%)	20 (4.48%)	24 (9.45%)	
Socioeconomic status (0–3)	1.43 (0.82)	1.44 (0.82)	1.40 (0.83)	0.558
Health literacy (1–12)	8.25 (2.27)	8.33 (2.20)	8.11 (2.38)	0.217
Digital health literacy (8–40)	28.36 (7.06)	28.70 (6.61)	27.76 (7.76)	0.090
Frequency of interaction with pharmacists/pharmacy technicians				
Never	160 (22.86%)	72 (16.14%)	88 (34.65%)	
Less than once a month	226 (32.29%)	148 (33.18%)	78 (30.71%)	
At least once a month	217 (31%)	152 (34.08%)	65 (25.59%)	
Twice to three times a month	71 (10.14%)	54 (12.11%)	17 (6.69%)	
More than three times per month	26 (3.71%)	20 (4.48%)	6 (2.36%)	
Any use of contraception method	232 (33.14%)	166 (37.22%)	66 (14.8%)	0.002
Previous use of oral contraceptives	129 (18.43%)	100 (22.42%)	29 (11.42%)	<0.001
Sexually active	274 (39.14%)	181 (40.58%)	93 (36.61%)	0.301
Interest in over-the-counter oral contraceptives (1–5) ^e^	2.28 (1.24)	3.02 (0.98)	1 (0)	<0.001
Contraception concern (0–4)	2.25 (0.68)	2.16 (0.66)	2.39 (0.70)	<0.001
Oral contraceptive knowledge (0–9)	4.08 (1.89)	4.11 (1.89)	4.03 (1.87)	0.559

^a^ Individuals who indicated that they were at least “slightly interested” were included. ^b^ Characteristics were compared between individuals who were interested in using over-the-counter oral contraceptives versus individuals who were not interested. ^c^ Three respondents had missing region responses. ^d^ Two respondents had missing rurality responses. ^e^ Out of 446 individuals who showed interest in using over-the-counter oral contraceptives, 161 individuals responded “slightly interested”, 165 responded “moderately interested”, 72 responded “very interested”, and 48 responded “extremely interested”.

**Table 2:** Oral contraceptive knowledge among subgroups of interest.

	Number of Individuals with Correct Answers (%)		
Oral Contraceptive Knowledge ^a^	All Included Individuals (*N* = 700)	Individuals with Interest in Using Over-The-Counter Oral Contraceptives (*n* = 446)	Individuals Who Were Not Interested in Using Over-The-Counter Oral Contraceptives (*n* = 254)
Total score (mean [standard deviation])	4.08 (1.89)	4.11 (1.89)	4.03 (1.87)
How often should someone take birth control pills?	436 (62.29%)	288 (64.57%)	148 (58.27%)
Which of the following methods has the lowest number of pregnancies expected, meaning the most effective?	328 (46.86%)	208 (46.64%)	120 (47.24%)
What is NOT the reason someone might use birth control pills?	322 (46%)	209 (46.86%)	113 (44.49%)
Jane is currently preventing pregnancy with the birth control pill. She has missed two pills. Today, she takes the last missed pill as soon as she remembers she forgot them. The pills won’t be effective for another 7 days from today.	403 (57.57%)	261 (58.52%)	142 (55.91%)
What is not a possible method of oral contraceptives preventing pregnancies?	218 (31.14%)	141 (31.61%)	77 (30.31%)
What are the different options currently on the market? Select all that apply.	325 (46.43%)	207 (46.41%)	118 (46.46%)
Oral birth control pills may increase the risk of breast cancer and cervical cancer.	402 (57.43%)	245 (54.93%)	157 (61.81%)
Which of the following is NOT the common side effects of the pill?	166 (23.71%)	117 (26.23%)	49 (19.29%)
For the health problem of leg pain or swelling, please select whether you would _____.	258 (36.86%)	159 (35.65%)	99 (38.98%)

^a^ The choices for each item and correct answers are available in Appendix A.

**Table 3:** Domains of oral contraceptive knowledge among subgroups of interest.

	Number of Individuals with Correct Answers (%) ^a^		
Oral Contraceptive Knowledge	All Included Individuals (*N* = 700)	Individuals with Interest in Using Over-The-Counter Oral Contraceptives (*n* = 446)	Individuals Who Were Not Interested in Using Over-The-Counter Oral Contraceptives (*n* = 254)
Side effects	65 (9.29%)	44 (9.87%)	21 (8.27%)
Mechanism of action	100 (14.29%)	69 (15.47%)	31 (12.2%)
Use	266 (38%)	173 (38.79%)	93 (36.61%)
Indications	322 (46%)	209 (46.86%)	113 (44.49%)
Efficacy	328 (46.86%)	208 (46.64%)	120 (47.24%)
Risk	402 (57.43%)	245 (54.93%)	157 (61.81%)

^a^ For domains involving two items, only those with both items correct were included.

**Table 4:** Association between individual characteristics and oral contraceptive knowledge.

*N* = 695	Adjusted Regression Estimate	Standard Error	*p*-Value
Age	−0.027	0.030	0.377
Race			
White	Reference		
African American	−0.591	0.162	<0.001
Hispanic	−0.619	0.197	0.002
Other (American Indian or Alaska, Asian, Native Hawaiian or other)	−0.317	0.200	0.113
Region			
Northeast	Reference		
Midwest	−0.085	0.217	0.693
South	−0.248	0.191	0.195
West	−0.195	0.218	0.372
Rurality			
Urban	Reference		
Suburban	−0.112	0.162	0.490
Rural	−0.186	0.183	0.309
Insurance status			
Covered under parent/guardian’s plan or private insurance	Reference		
Public insurance	−0.350	0.148	0.019
Uninsured	−0.594	0.192	0.002
Marital status			
Never married	Reference		
Married	−0.222	0.267	0.406
Other (divorced, separated, widowed, prefer not to say)	−0.288	0.173	0.097
Highest education attained			
High school or less	Reference		
Some college or Bachelor degree	0.157	0.182	0.387
Advanced degree	−0.499	0.408	0.221
Prefer not to say	0.292	0.273	0.285
Socioeconomic status	−0.039	0.079	0.624
Health literacy	0.125	0.030	<0.001
Digital health literacy	0.019	0.010	0.052
Frequency of interaction with pharmacists/pharmacy technicians	−0.211	0.064	0.001
Any use of contraception method	0.580	0.201	0.004
Previous use of oral contraceptives	0.549	0.226	0.016
Sexually active	0.622	0.149	<0.001
Interest in over-the-counter oral contraceptives	0.014	0.136	0.916
Contraception concern	0.293	0.096	0.002

## Data Availability

The data presented in this study are available on request from the corresponding author due to the inclusion of minors.

## Appendix A

**Table A1:** Oral contraceptive knowledge items.

Items	Choices (Correct Answers Are Underlined)	Domains
How often should someone take birth control pills?	Once a day Once a week Once a month Once 6 months	Use
Which of the following methods has the lowest number of pregnancies expected, meaning the most effective?	Intrauterine device (IUD) that is inserted and removed by a health care provider Oral birth control pills Software application for contraception Male condom Cervical cap with spermicide	Efficacy
What is NOT the reason someone might use birth control pills?	To prevent sexually transmitted diseases To regulate your menstruation cycle To prevent pregnancy To alleviate acne	Indication
Jane is currently preventing pregnancy with the birth control pill. She has missed two pills. Today, she takes the last missed pill as soon as she remembers she forgot them. The pills won’t be effective for another 7 days from today.	Yes No	Use
What is not a possible method of oral contraceptives preventing pregnancies?	Prevention of ovulation Thickening the mucus Killing the sperm Thinning the endometrium (inner line of uterus)	Mechanism of action
What are the different options currently on the market? Select all that apply.	Pills containing a combination of progesterone and estrogen Pills containing a combination of testosterone and estrogen Pills only containing estrogens Pills only containing testosterone Pills only containing progesterone	Mechanism of action
Oral birth control pills may increase the risk of breast cancer and cervical cancer.	Yes No	Risk
Which of the following is NOT the common side effects of the pill?	Intermenstrual spotting Nausea Breast tenderness Diarrhea Headaches and migraine Weight gain Not sure	Side effects
For the health problem of leg pain or swelling, please select whether you would _______	Continue taking the pill; call the doctor immediately Stop taking the pill; call the doctor immediately Continue taking the pill; plan to discuss with the doctor Stop taking the pill; plan to discuss with the doctor Not sure	Side effects
